# Intergenerational continuity of protective parenting practices in Dhaka, Bangladesh

**DOI:** 10.1371/journal.pone.0300160

**Published:** 2025-02-28

**Authors:** Umme Habiba Jasmine, Mzikazi Nduna, Busisiwe Nkala-Dlamini

**Affiliations:** 1 Department of Psychology, University of the Witwatersrand, Johannesburg, South Africa; 2 AVREQ, University of Stellenbosch, Stellenbosch, South Africa; 3 Department of Social Work, University of the Witwatersrand, Johannesburg, South Africa; IIPS: International Institute for Population Sciences, INDIA

## Abstract

Intergenerational transmission of parenting practices is a new dimension of research in Bangladesh. This study used a social cognitive theory framework along with a theory of urbanization and a theory of modernization to examine the continuity of protective parenting practices across two generations. A cross-sectional hermeneutic phenomenological study design was followed from a social constructivist paradigm. Eleven maternal grandmothers (G1) and 11 mothers (G2) were purposively selected from Mirpur, Dhaka, Bangladesh. Data were collected through semi-structured, in-depth interviews. The mothers learned protective parenting practices from the grandmother generation through direct instruction, experience, observation, and modeling within an interaction of various social elements. Protective parenting practices were found to be essential and persisted with some modifications despite modernization. Based on the findings, a process model of intergenerational continuity of parenting practices has been offered, which depicts intergenerational learning within a transitional social context.

## Introduction

### Intergenerational continuity of parenting

Intergenerational (IG) continuity of parenting practices delves into sharing parenting behaviors, values, and techniques across generations. Researchers started intergenerational parenting studies based on assumptions that parenting knowledge, practices, and styles are passed down to the following generations, and the following generations mimic parenting behaviors of their parents knowingly or unknowingly. IG parenting studies were started with retrospective data based on these appealing intuitions. However, because of the limitations of recall biases and people’s tendency to validate their parenting behavior, researchers initiated prospective IG studies in the 1990s [1]. Terms such as intergenerational transmission, intergenerational continuity-discontinuity, and intergenerational stability-instability have been used in IG studies that communicate similar meanings with slight differences. Van Ijzendoorn [2, pp 76-77] defined intergenerational transmission of parenting as “the process through which purposively or unintendedly an earlier generation psychologically influences parenting attitudes and behavior of the next generation.” However, it extends beyond individual families, as societal, cultural, and environmental factors play vital roles in this process [3,4]. Intergenerational stability denotes similarities in rank order, whereas intergenerational continuities denote similarities in mean levels of behaviors across generations [1]. However, the term intergenerational continuity-discontinuity appeals to us in this fundamental study in the Bangladeshi context because we believe that the exploration of similarities and differences across generations should come first before stability rank order. We consider that intergenerational continuity is an active process where, consciously or unconsciously, both generations play an active role in the process.

Child developmentalists have been intrigued by the question, “Why do parents parent in the way they do?” [5,6]. Researchers determined to answer this question focusing on IG transmission/ continuity of parenting practices. Numerous studies investigating IG parenting have provided supporting evidence using multigenerational samples [2,7–10].

A literature review by Kerr and Capaldi [1] reported the paucity of IG parenting studies, and that the available studies mostly focused on problem outcomes such as psychopathology and substance abuse other than parenting itself. There are countless studies on the intergenerational transmission of corporal punishment and abusive and harsh parenting [5,11–17], while other parenting aspects received less attention. A couple of studies are found in the area of intergenerational transmission of attachment [18,19], parental monitoring [11], parental rejection [20], emotional warmth and acceptance [21], constructive parenting [22], and initial reaction [23]. Yet, exploration of intergenerational continuity of specific parenting dimensions that comprise a set of parenting practices is limited. As such, research on the intergenerational continuity of *protective* parenting dimension is limited except for the practice of parental monitoring, which is one of the practices of protective parenting dimension. While researchers in Western countries have examined intergenerational parenting transmission; parenting in general, protective parenting practices in particular, remains understudied in Bangladesh [24]. This study addresses research gaps by exploring the intergenerational continuity of protective parenting practices among urban-dwelling middle-class Bangladeshi mothers.

Modernization effects, economic and historical changes in society, culture, and the external world affected the context, attitude toward certain parenting behaviors, and outcomes [1]. After approaching millennium two, the number of women joining the workforce increased remarkably, which empowered the current generation of mothers in many ways, more importantly, financially and in decision-making. However, it also caused a mother’s absence in childcare for a long period of a day. This indicated a possibility of intergenerational change in parenting practices and sharing parenting responsibilities with the father and/ or other family members. Increased substance usage in society, availability of contraceptives to decrease teenage pregnancy and sexually transmitted diseases, availability of cell phones, and parents’ and children’s exposure to the internet and social media, have a bearing on real and perceived threats for children [1]. This appeals to different approaches to parenting while fundamental parenting concepts and practices could remain similar.

The impact of modernization might cause the dynamics of intergenerational continuity of parenting practice to be more complex than ever before. Traditional parenting knowledge and cultural practices, that conserve distinct customs and contribute to building resilience [25], might be lost if not passed over through media, mainstream education, and documentation [26]. Besides, intergenerational continuity of parenting practices in a shared communal culture like in Bangladesh might not be the same as in individualistic Western societies. In-depth exploration would inform the planning of parenting and child development programs for Bangladeshi communities to promote positive outcomes while reducing the negatives. In-depth exploration would also suggest effective modes of sharing and preserving parenting knowledge and would enrich Indigenous and global parenting literature.

### Theoretical Framework

Psychologists and anthropologists regard social learning theory as a universal learning mechanism for cultural knowledge and practices [27]. Therefore, social learning theory is commonly discussed as a key mechanism of intergenerational continuity of parenting behaviors [9,17,28]. However, the social cognitive framework [29], a later version of social learning theory, was most suitable for this study. In addition to incorporating all the learning mechanisms, such as observation, direct instruction, and modeling, the social cognitive theory emphasizes a person’s cognitive and decision-making aspects in learning and practices.

Although we started this study with a social cognitive framework, it was found inadequate to explain the data that emerged in the context of urbanization and modernization. Therefore, Wirth’s [30] theory of urbanization, and Inkeles and Smith’s [31] theory of modern man were added to the theoretical framework. The functionalist perspective of the theory of urbanization was used, which suggests that urbanization brings both positive and negative outcomes, and the residents respond to the effects with greater tolerance and creativity. The participants’ creativity in responding to the urbanization-induced insecurity challenges in Dhaka city was addressed with this theory of urbanization.

The lens of the theory of the modern man [31] was used during data analysis, which states that a modern person- 1) keeps themselves updated about major events and news, 2) believes that their actions might affect his life course, and that his active efforts can improve his as well as his family conditions. 3). is independent of the traditional authority sources, and 4) is open to novel experiences. The theory also suggests that a modern person uses their choice and decisions and does not ‘soak up’ everything modernization brings [p 82]. This perspective aligns well with the cognitive aspect of social cognitive theory [29]. The emphasis on a person’s free choice and decision-making skills of modernization theory also aligns well with the theory of urbanization [30], which emphasizes a person’s creativity in responding to the effect of urbanization.

### Study site and study population

This study focused on the parenting practices of the middle-class population of Dhaka, the capital of Bangladesh. The following matters were considered for choosing the middle class as the study population. The lives of the middle class generally center around their family; preparing their children for a bright future is their top priority. History suggests that the middle-class population played irrefutable roles in socio-cultural activities, intellectualism, political change, and leadership [32]. Besides, the middle class constitutes a large portion (25%) of the entire population in Bangladesh [32].

The study field, Dhaka, is densely populated, accommodating around 20 million people [33]. As the center of education, administration, and financial opportunities [34], this cosmopolitan city attracts around 30,000- 40,000 inland migrants every year [35]. It hosts people from various subcultures of Bengali culture. Consequently, we considered Dhaka an opportune setting to obtain enriched data on protective parenting.

In Dhaka city, huge-scale urbanization started in the 1990s when a large influx of inner migration and housing for the middle-class population started there. A second wave of urbanization took place in the 2000s with remarkable development in residential and commercial aspects. The urbanization process configured a new cultural identity for the migrants and tarnished the inherent nature of the community [34]. Sultana [36] reported that children in Dhaka city grew up in isolation inside homes with no interaction and not knowing the closest neighbors. This impacted their social bonding, social values, and responsibilities. Gradually, Dhaka has become a city of insecurity and crime. A rise in drug use, kidnapping, and violence against women was reported in 2006 [37], which is also valid to date [38]. The study also reported increasing loss of trust and loss of mutual understanding among the residents.

In the last two decades, the Shahbagh, Uttara, Mirpur, and Mohammadpur areas of Dhaka city have been primarily occupied by middle-class people. Among these areas, Mirpur was selected as the study location based on one primary condition: greater access to the study site. The first author grew up in Mirpur and was an insider by residence and lifestyle. This helped to achieve trust and access in the community for data collection. Besides, around 47 percent of the population in Mirpur belongs to the middle class [34]. Around 45% of the total income of Mirpur residents comes from fixed jobs [34]. This helps them plan for the future of their children and families.

A threshold of USD 2-3 per capita per day income is considered to be included in the middle class [39]. A survey on the emerging middle-income class in Bangladesh revealed that the monthly family expenditure of 85% of middle-class households was between BDT 30K to 99K, 15% had BDT 100K to 250K, and a negligible number of households had BDT 250K [40]. The family income stated in Table 1 indicates that G2 participants belonged to the middle class. The family income during children’s school-going stages of G1 participants was not collected because that would not explain their former socioeconomic status now. However, none of the participants reported or indicated poverty, financial hardship, or financial abundance while sharing their parenting experiences.

**Table 1:** Socio-demographic characteristics of participants.

Dyads	Type of Participants	Pseudonym	Age in year	Highest Education obtained	Profession	Monthly Family Income (BDT) and sources	Number of children	Age range of children
Dyad # One	Mother	Panna	42yr	Masters	Housewife	50,000; Husband’s business	1	9 yrs
	Grandmother	Tara	56yrs	Below SSC^*^	Housewife	100000; rent and Husband’s business	4	19-34yrs
Dyad # Two	Mother	Dali	50yrs	HSC^**^	Housewife	48,000; rent and Husband’s job	2	18-29yrs
	Grandmother	Hena	69yrs	Grade 5	Housewife	35,000; Rent	4	33-52yrs
Dyad # Three	Mother	Shirin	49yrs	Masters	Housewife	40,000; Husband’s job	2	9-18yrs
	Grandmother	Fatima	60yrs	Matric	Housewife	40,000; rent and Husband’s pension	3	40-52yrs
Dyad # Four	Mother	Tonu	37yrs	Masters	Housewife	35,000; Husband’s job	1	12yrs
	Grandmother	Mina	62yrs	Matric	Housewife	25,000; Rent	3	29-41yrs
Dyad # Five	Mother	Shoshi	48yrs	HSC	Housewife	50,000; Husband’s business	2	17yrs (twin)
	Grandmother	Rehana	66yrs	Grade 8	Housewife	100,000; rent and Husband’s pension	2	45-48yrs
Dyad # Six	Mother	Pinky	30yrs	Masters	Housewife	50,000; Husband’s job	2	1-7yrs
	Grandmother	Jahan	49yrs	SSC	Housewife	40,000; Rent	3	19-30yrs
Dyad # Seven	Mother	Piu	28yrs	HSC	Housewife	45,000; Husband’s job	2	3.5-8.5yrs
	Grandmother	Amena	48yrs	Grade 5	Housewife	32,000; Husband’s job	3	17-28yrs
Dyad # Eight	Mother	Farida	46yrs	Masters	Advocate	85,000; rent, profession, and husband’s business	2	14-19yrs
	Grandmother	Jobaida	80yrs	Grade 5	Housewife	35,000; Rent	9	Not available
Dyad # Nine	Mother	Charu	48yrs	Masters	Teacher	70,000; rent, profession and Husband’s job	2	12-21yrs
	Grandmother	Khadija	66yrs	<Grade 5	Housewife	25,000; Rent	2	32-48yrs
Dyad # Ten	Mother	Dina	34yrs	Masters	Teacher	100,000; profession and Husband’s business	2	6-12yrs
	Grandmother	Sharifa	51yrs	Below Matric	Housewife	50,000; Husband’s job/ pension	4	22-34yrs
Dyad # Eleven	Mother	Jhumjhum	36yrs	Masters	Banker	80,000; job and Husband’s job	1	12yrs
	Grandmother	Kahi	57yrs	Below Matric	Housewife	120,000; rent and Son’s contribution	5	22-38yrs

## Method

This study used a social constructivist paradigm and a qualitative research approach. A cross-sectional hermeneutic phenomenological research design was followed. The study aimed to understand the meaning and process of continuity of protective parenting practices constructed in the Bangladeshi cultural context from the experiences of two generations of mothers. Purposive snowball sampling was used to recruit 11 mother-maternal grandmother dyads of participants from Mirpur in Dhaka, Bangladesh. Data was collected through semi-structured, in-depth interviews. Data was analyzed following a hermeneutic circle method.

### Ethical considerations

Ethics approval was obtained from the Human Research Ethics Committee (non-medical) of the University of the Witwatersrand, South Africa (protocol number: H16/06/38), and the Department of Clinical Psychology Ethics Committee, University of Dhaka, Bangladesh (project number: PH160603). For better communication with participants, the Bengali version of the participant information sheet and informed consent form were used. Besides, the first author, who was also the interviewer, explained the participant information sheet and informed consent form in Bengali for the participants’ better understanding. Written informed consent was collected from all but one G1 participant (Khadija), who gave verbal consent because of having no trust in written consent.

The ethics application stated that the interviews would be conducted in private. Yet, the plan changed following the pilot study when the researchers discovered a shared sense of confidentiality in the population as part of a communal culture. Additionally, conducting interviews closed door affected rapport and brought family members’ disappointments towards both the participants and the researcher. Participants also did not agree that closed-door privacy was necessary. Therefore, the following interviews were conducted keeping the door semi-closed. A semi-closed door communicated a sense of privacy and allowed interested family members to enter the room when deemed necessary.

### Inclusivity in global research

Additional information regarding the ethical, cultural, and scientific considerations specific to inclusivity in global research is included in the Supporting Information (S1 Checklist).

### Participant selection and data collection

The parenting practices of two generations of Bangladeshi mothers were explored using semi-structured in-depth interviews of 22 purposively selected participants of 11 mothers (*second generation; also referred to as G2*) and 11 maternal grandmothers (*first generation or G1*). The participants were selected based on three criteria: a) the mothers (G2) with at least one grade 1 to grade 12 school-going child, b) the maternal grandmother (G1) be accessible and fit for an in-depth interview, and c) the participants with not developmentally or mentally challenged children. The grandmothers were the biological mothers or primary caregivers of the mothers. Participants were assigned pseudonyms, and the recorded interviews were translated, transliterated [41], and transcribed [42] into English. We emphasized cultural equivalence [43] in the process. NVivo 10 software was used for coding and data management.

Along with data collection, the first author occasionally wrote field notes and memos, which helped with data analysis and interpretations. The field notes were about their experience, the environment of the interview sessions, and relevant and noticeable information. The memo was about their biases, hunches, interpretations, hypotheses, and connections among the codes, ideas, and discussions. Table 1 depicts the participants’ socio-demographic background.

### Data analysis

Inductive data analysis was conducted using a hermeneutic circle. UHJ repeatedly read each transcript and the field notes to understand the stories’ parts and wholes and generated initial codes. UHJ was attentive to the participants’ altered perceptions and interpretations while reading the texts. They applied axial coding to categorize the initial codes based on functional connectedness and perspectives. UHJ read and re-read the code categories, texts, research objectives, and research questions to generate broader themes and sub-themes and to interpret participants’ lived experiences. Initial themes were generated. Then, the initial themes were refined based on internal homogeneity and external heterogeneity [44]. The protective parenting theme was divided into four sub-themes: practices, beliefs, learning, and influencing elements. The data on each sub-theme were compared and contrasted between G1 and G2. This procedure was carried out multiple times till the manuscript was finalized.

MN verified the coding, themes, and analysis. UHJ’s knowledge and experience as an insider, as well as the literature on culture and traditions, helped contextualize and interpret the data. MN and BND checked for personal biases throughout. Accordingly, we were persistently aware of researcher biases and sensitive to the text’s ‘alterity’ [45, pp 268-273].

Data triangulation was achieved by collecting data from participant dyads of two consecutive generations. Their inputs supplemented and validated each other’s data without sharing information. The credibility of this study was achieved in three phases. During interviews, open-ended questions were used to avoid imposition of the researcher’s meaning on data. After translation and transcription, a language editor matched the Bengali interview audio with the English transcript. Finally, during the data analysis phase, the findings and interpretations were repeatedly checked against the raw data to maintain ‘referential adequacy’ [46, p 301]. Besides, descriptive notes were taken during the research process, member checking was conducted, and peer debriefing was also maintained from the formation of the questionnaire up to the end of writing this report. Confirmability of this study was achieved by an audit trail and inquiry audit, which were conducted by the second and third researchers, respectively.

## Findings

This study aimed to understand the patterns, sources, meaning, and process of continuity of protective parenting practices based on the experiences of two generations of mothers. The findings suggest that protective parenting practices are learned through parental coaching, modeling, experience, vicarious learning, and observations within an interaction of social and cultural elements. Sometimes, mothers spontaneously internalized certain parenting practices and claimed that those were instinctual.

### Observational and vicarious learning

The participating mothers (G2) acknowledged learning about chaperoning and saving money for their children’s future through observations of their mothers’ (G1) practices. Chaperonage was present in both generations’ parenting practices, but G2 mothers exhibited changes and variations shaped by evolving safety concerns in their social context.

Four mothers (G2) affirmed that they witnessed their mothers (G1) chaperone their (G2) younger siblings and identified the strength of the practice in revealing their younger siblings’ secrets and deceptions. Thus, chaperonage appeared to be a powerful tool for mothers in protecting children from derailment. For example, Piu (28-year-old mother, G2) described how chaperonage helped in stopping her sister’s deceptive behavior with their parents,

My sister used to return home alone after school; there were enough opportunities for deception. She used to return one to two hours after school and say, “School finished now!” Once, my mother went to fetch her and found her playing. My father told [my mother], “… If you started fetching her earlier, she could not have deceived us this whole time.”

Thus, Piu vicariously learned about the usefulness of chaperonage and practiced it to prevent her children from being deceitful and to monitor their friend circles. Dali (50-year-old mother, G2) also reported a similar learning experience.

Parents’ responsibility to ensure their children’s financial security was also learned from observing their mothers’ practices of saving money for their children’s future. Jhumjhum (34-year-old mother, G2) shared that her parents (G1) spent money on her and her siblings’ education and weddings, and she was also saving for her only son’s (G3) education and wedding program, following her parents’ example. Jhumjhum said,

My brothers’ weddings were gorgeous, and my parents spent a lot of money on their weddings… So, I have planned to arrange my son’s wedding more luxuriously … I have started to make gold ornaments bit-by-bit… because I cannot buy those all at once. I want to give many things to my daughter-in-law … I will make her gorgeous.

Charu (a 48-year-old mother, G2) also planned to finance her daughters’ weddings, as her mother Khadija (a 66-year-old grandmother, G1) did for her. Vicarious learning and observational learning played a critical role in adopting mothers’ practices of chaperonage and ensuring financial security for children, thus facilitating their continuity intergenerationally.

### Childhood experiences

By comparing the reports of the G1 and G2 participants, practices such as training children to share their daily whereabouts, monitoring their social circle, and imposing gender boundaries were found common in both generations and often with slight modifications in the mother generation (G2). This indicates the carryover of certain practices from one’s own childhood experiences.

Dali’s (50-year-old mother, G2) mother, Hena (G1), trained her to report her whereabouts to parents. After becoming a mother, she trained her children to do the same and continued the practice until they were adults.

*“*Never go anywhere without informing me!” My mother probably told me once when I was admitted to the school. But that word rang in my ears the longer I continued my studies. [I used to tell myself], “I cannot go anywhere without informing my mother!” My daughter follows it the exact way.

Developing a child’s practice of updating parents about their whereabouts appeared to be a form of monitoring children from a distance, and an attempt to control children’s movement. This practice was also an effort to generate self-control within their children by keeping them mindful of their accountability and of their parents’ constant monitoring.

All the participants from both generations valued gender boundaries unexceptionally for their capacity to protect girls from sexual abuse and harassment by limiting girls’ movement outside the home and by reducing girls’ exposure to men. Daughters were prohibited from talking to male relatives in private, visiting neighbors alone, or sleeping over without the mother. Fatima (60-year-old grandmother, G1) validated that restrictions to girls’ free movement were motivated by Islamic values. Fatima’s parents were strict religious practitioners and did not let her spend time with her male cousins alone. Although she was not a strict religious practitioner, she followed the same practice and did not allow her daughter Shirin (49-year-old mother, G2) to talk much with male cousins. Fatima said,

My father’s family was very conservative and Islamic-minded… My father’s family taught me to behave differently towards a male and a female. Girls in my father’s family do not freely interact with other boys. When my daughter got older, I did not allow my nephews or other relatives to stay in my house. Also, I did not [let my daughter stay] at their houses. [My attitude was], “You have come for a visit, okay visit, have some meal and leave! You will stay at my home… chat with my daughter- that will not happen!” I didn’t allow that.

Schooling in co-education, Shirin’s (G2) daughter (G3) had more access to male classmates. Yet, Shirin maintained similar restrictions, did not allow their own daughter to interact with men or male cousins, and always interviewed if she ever talked to them. Shirin said,

I warned her about boys and related issues. I told her “… make sure that I do not catch you going out with a boy” … I try to make her understand these sometimes. She says, “No, mother, I don’t [have interest] in those kinds of stuff” … if she gets a phone call, [she says], “My female friends call me. No boy calls on my phone.” … if she takes too long on the phone, her father scolds her, “Who are you talking to?” Then she’d reply, “No… my friend called, I’m talking to (.) my friend.”

Cell phones and internet access have become easy in the modern world, and that appears to have created anxiety among Bangladeshi parents about losing a grip over children and about children exceeding their boundaries. Therefore, the G2 participants had to extend monitoring of their child’s interaction with their opposite-gender relatives or friends. Although in some cultures, first cousins are considered close as siblings, and marriage is prohibited among them [47,48], this is not the case in Bangladeshi culture. This might have prompted the participants to protect their daughters from male cousins in the same way they would from any non-relative male. Each mother undertook different strategies to monitor their children’s friendships. Khadija (66-year-old grandmother, G1) and Jobaida (80-year-old grandmother, G1) used to host their children’s friends and establish connections with their parents. Khadija’s daughter Charu (48-year-old mother, G2) and Jobaida’s daughter Farida (46-year-old mother, G2) similarly hosted and quizzed their adolescent and young adult children’s friends about their family backgrounds, to assess their suitability as friends. Farida extended the monitoring strategy by befriending her children’s friends to control their activities indirectly because her children were not very obedient, and she wanted to safeguard them from negative influences. Farida said,

My mother knew my friends… They used to visit our house. My mother and all my friends’ mothers would think that… their daughters’ friends were like their own daughters… The mothers had connections among themselves of their own… I asked our sons to bring over their friends, and I would investigate what kind of company they were and what their families were… My younger son, Prince, is fond of Kiron, my elder son’s friend… I ask Kiron, “Talk to Prince... Tell him to hang out only with Tutul and Mubin.”

Both the G1 and G2 mothers’ concerns and efforts regarding children’s friendships reflect their conviction in a Bengali proverb that says, *‘A bad association can cause an iron rod to float,’* which means that a good friend positively influences lives, and a bad friend influences negatively. Childhood experience armed the participants with some protective parenting practices, and the G2 participants adapted or extended those practices based on their assessment of the current social safety context and their children’s characteristics to avert potential harm to their children.

### Direct guidance and instructions about protecting children

Maternal grandmothers (G1) were found to have a considerable influence on teaching parenting practices. Mothers (G2) specified maternal (G1) guidance and direct instructions as a source of learning about protective parenting practices such as gender boundaries, restricting children’s movements, and traditional rituals.

Four mothers reported being explicitly instructed by their mothers (G1) to accompany their daughters when they visit friends. Grandmothers (G1) expanded their role by guiding mothers (G2) on safety measures for their grandchildren (G3). The safety measures included- not leaving daughters at home alone, restricting their children’s movements, and ensuring that children did not stay outdoors in the evening. Khadija (66-year-old grandmother, G1) advised her daughter Charu (48-year-old mother, G2),

I will not tolerate my granddaughters associating with bad children, and they must not go out in the evening. They must return home before Maghrib (sunset time). This era is truly very bad. *You* should not let your children go out alone. As girls grow up, it is inappropriate to leave them alone. It is a bad time. Instead of studying, they may get into relationships with boys… do not even leave them home alone.

The participating mothers (G2) mentioned that their mothers (G1) also educated them about the traditional beliefs, customs, and child protective rituals against evil spirits. The reported rituals were restrictions in attending to groceries soon after bringing them home, not bringing fish or meat home after evening, and cleansing fried and sweet food by whirling on the fire. Thus, the grandmothers’ generation (G1) played the role of responsible guardians. Kahi (57-year-old grandmother, G1) reported learning pregnancy-related customs and rituals from her mother and elders and instructed her daughter Jhumjhum (34-year-old mother, G2) to follow those during pregnancy,

Djinn stays with raw fish, meat, and chicken… After others take care of groceries, you will get close to that half an hour later… Do not eat those uncleansed foods immediately!... If it is a fruit or fried snack, whirl it on the fire, nicely! Otherwise, it would be harmful to both you and the fetus.

The G2 mother, Jhumjhum, was highly educated, with a Master’s degree. Yet, she strongly believed in traditions and chose not to give up tradition- and culture-supported practices in child protection. Both generations of participants expressed honor and trust in their mothers and identified them as important agents in their process of learning protective parenting. Sometimes, the mothers (G1) modeled those practices, and sometimes, they instructed their daughters (G2) before and after childbirth.

### Other sources of learning

Participants mentioned other learning opportunities besides learning from their mothers. G2 participants sometimes received advice and directions from fathers and husbands about escorting children to school. Primarily driven by safety concerns, family members supported and encouraged protective parenting practices. Elders in the community, local proverbs, and urban legends reinforced practices such as not praising children, selecting friends from good families, and not giving money to young children.

Besides the direct instructions from the grandmothers (G1), the significance of chaperonage children was emphasized by the mothers’ peer groups, media reports, and other family members. Sometimes, children’s behavior and actions were reported to influence protective parenting. Shoshi (47-year-old mother, G2) and Farida (46-year-old mother, G2) sometimes found their sons lying and engaging in culturally disapproved behavior, and they had to be creative to extend parental protection. Shoshi explained, “I called to check whether he (son) went to the coaching center truly… [I become sure of it] by listening to the teacher’s voice and the children’s voice. Boys are not trustworthy, (.) understand?”

Children’s ‘inappropriate’ conduct thus prompted the adoption of extended monitoring methods, including frequent phone calls, befriending children and their friends to control children’s whereabouts, and having access to daily updates. Extended monitoring strategies have been reported to bring some positive changes in children’s conduct. However, the extent of mutual influence cannot be reported here as this study did not explore children’s accounts.

Access to global media exposed children to socially unacceptable content, resulting in the development of new protective parenting strategies, which were absent and irrelevant in G1 participants’ childrearing time. Tonu (37-year-old mother, G2) explained how technology has grounded extended monitoring strategy,

I do not let her (daughter) use mobile phones at night. The TV is in my bedroom, so she watches TV there… She doesn’t have a personal tab because if I give her a tab, I will not be able to monitor her… At night… she may chat on Facebook… These days… there is a lot of adult content on the internet she may come across and watch. One day, it will interfere with her studies, and her focus will be diverted. We want to guide her until A-level so that her base strengthens.

Participants had heightened concern about children’s media access and that called for their creativity to put a balance between access to modern technology and preserving local traditions and cultural norms. Mothers (G2) also engaged in open conversations with children and were vigilant of warnings of substance abuse, while the mothers (G2) reported an increase in the availability and misuse of substances in recent years.

**Fig 1:**
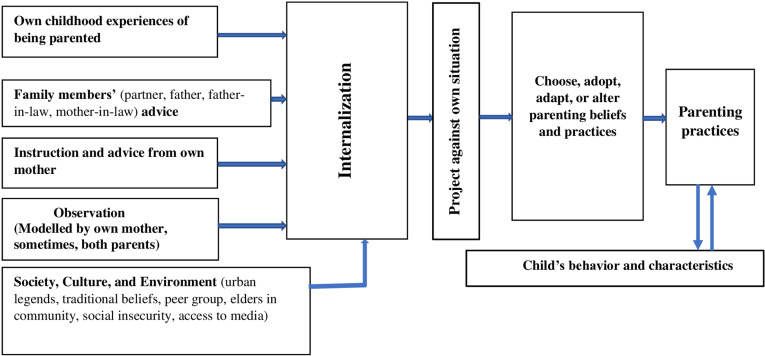
Process model of intergenerational continuity of protective parenting practices.

Thus, the intergenerational continuity of protective parenting practices occurred through an interplay of direct instruction, childhood experience, observations of mothers’ practices, and other learning opportunities in the context of urbanization and modernization in a traditional communal society. Based on these findings, we propose a process model of intergenerational continuity of protective parenting practices in Fig 1.

According to this intergenerational continuity process model, learning of protective parenting practices starts at home within a complex interaction with social endeavors. A person carries their childhood experience of being parented. Besides, while growing up, they observed their parents’ protective practices and parents carrying protective values. After becoming a parent, the person often receives various parenting advice and instructions, mostly from their mother, and also from a partner, parents-in-law, and extended family members. As a member of society, a parent interacts with cultural and traditional knowledge, practices, and beliefs, urban legends, peer groups, seniors, community members, emerging technological and global changes, and various aspects of the community, including security. A person continuously internalizes knowledge and practices from all these resources. When they raise their children, they project their knowledge onto their individual situations. Using their rational thinking, openness, knowledge, and traditional, emotional, and moral values, a parent then chooses which parenting practices to implement with their children and which ones they would modify or alter according to current needs and situational demands. Through this process, a parent develops and determines the parenting practices they would implement in raising their children. Children’s behavior and characteristics remain as live modifiers in the process, which explains why a parent’s parenting practices might differ for two children in the same household.

## Discussion

This study explored the continuity of protective parenting practices between two generations of Bangladeshi mothers. Supporting the social cognitive theory [29], the intergenerational continuity of protective parenting practices took place through an interaction of a set of learning opportunities and contextual factors such as negative changes in social security, access to media, cultural practices, advice, and instruction from family and members of the community. The findings are in line with Patterson’s [49] emphasis that intergenerational continuity of parenting takes place through modeling. The findings are supported by Belsky’s [50] process models of determinants of parenting, which explain that parents’ parenting behaviors are developed from parents’ experience of growing up and the personality of the child.

A couple of intergenerational transmission or continuity of parenting models are found in literature, which are predominantly based on parenting mediating and moderating factors, parents’ adjustment, and adult and child characteristics and behaviors [1,51]. Limited learning principles are found to be applied in the intergenerational parenting models in the literature, for example, in Patterson’s and Belsky’s models. However, our proposed intergenerational continuity process model fits very well with all the learning principles. Our model is powerful in addressing a person’s internalization and application of judgment and decision-making in determining and shaping parenting practices. Besides, it addresses individual children’s characteristics in determining the different parenting practices of the same parent to different children in the same household and context. This combination makes this model unique.

G2 mothers raised their children in Dhaka City, where a predominantly individualistic upbringing was practiced. Despite that, combined family loyalty still existed, which was evident in the grandmothers’ dedication to securing the future and safety of their grandchildren. Modifications in the protective practices, specifically, in chaperonage, monitoring, and spouse selection, among the G2 generation were evident. This reflects that the historical and secular changes influenced the parenting of Bangladeshi mothers. A change has been regarded as normal by Mohsin [52, p 627], suggesting that “continuity of a certain practice, belief or norm happens, but that too cannot manifest in its original form.” Mohsin [52] pinpointed elements such as science and technology, the interaction of ideas, education, media, economic conditions, and knowledge advancement, as contributors to this change, which is consistent with the findings of this study.

Although the learning part of the intergenerational continuity of protective parenting practices can be explained by social cognitive theory, the explanation of the changes in practices calls for Wirth’s [30] functionalist perspective of the theory of urbanization and Inkeles and Smith’s [31] theory of modern man. In support of Wirth’s [30] theory, this study revealed weakened social bonds, lack of neighborhood watch, development of impersonality along with structural development and opportunities, access to advanced technologies, residents’ worries, and creatively finding ways to protect children from being victims of city-centered crimes. Aligned with Inkeles and Smith’s [31] theory of modern man, this study revealed all four characteristics of a modern person among the participants, which indicates participants embracing modernization while preserving traditional values. Firstly, the participants constantly kept themselves updated about the major events in the community through newspapers, television, and the exchange of information. Secondly, the negative incidents generated insecurity feelings in them. It did not make them helpless; rather, they demonstrated a sense of self-efficacy through forming the practice of chaperonage, extended monitoring of children’s use of technology, and using cell phones to monitor children’s whereabouts to increase their sense of security. Thirdly, although the participants continued traditional rituals learned from the earlier generation because they chose not to abandon those, they were still open to gathering and selectively using knowledge from books, television, the internet, and social media. Fourthly, the G2 participants were open to change and to new experiences. They used new technologies, let children use technologies, and were open to children’s use of agencies in a spouse selection within certain limits instead of the total arranged marriage they had experienced. The cognitive and decision-making aspects of the social cognitive theoretical framework [29] and the third proposition of Inkeles and Smith’s [31] modernization theory can explain why G2 mothers followed some traditional parenting practices while living in the modern world. They can also explain why G2 mothers practiced the protective parenting behaviors they had learned from G1 mothers despite having access to updated knowledge through media, the Internet, and books.

The continuity of protective parenting practices in this study aligns with reciprocal determinism [29] within the interaction of environmental factors, open access to information and media, personal experiences, and observational learning. Studies investigating the intergenerational transmission of parenting (see [2]) have used Crittenden’s [53] three transmission models: 1) Observational learning of a parent interacting with other children, 2) experiences of being parented, and 3) parental coaching to the next generation. Crittenden’s models, which were based on social learning theory, were found to be inadequate in explaining this study’s findings, as they did not explain the roles of social context, the impact of technological development and globalization, child’s behavior, and how other environmental factors influence the process.

The findings on child monitoring strategies addressed Bakermans-Kranenburg and van IJzendoorn’s [54] question, “What strategies do modern parents use to protect their offspring: tend-and-befriend (use of social relationships for protection) or tend-and-defend (aggression against the threatening stimulus)?”. In this study, both generations of mothers (G1 and G2) used the tend-and-befriend strategy.

In the Indian subcontinent, maternal grandmothers often support new mothers with childcare [55,56] and shape parenting practices in line with their beliefs, culture, and religion. Although new mothers typically live with their in-laws while raising their children [57], none of the participants referred to the paternal grandmothers as a source of learning. It could be said that the maternal grandmother holds a distinct role in transferring parenting practices to the mother’s generation. However, at this stage, it is difficult to definitively conclude that due to methodological reasons as the study did not include paternal grandparents. Future studies could consider including paternal grandmothers, grandfathers from both sides, fathers, and other members of the family as part of the communal culture to obtain a comprehensive picture of the intergenerational continuity of protective parenting practices. Besides, future studies collecting children’s feedback on protective parenting practices, socio-cultural contexts, and intergenerational continuity will contribute to revealing the overall scenario.

The study findings place maternal grandmothers as the most mentioned considerable contributor to intergenerational continuity, where social context and technological development paved the grounds for protective parenting practices. The G2 mothers adopted new protective parenting measures to cope with threats generated by urbanization and modernization. Local proverbs, child’s behavior, social norms, elders in the community, peer groups, and other family members were also identified to contribute to the process. The process model of intergenerational continuity of parenting practices, sources of learning, and their interaction within the transitional social context are this study’s scholarly contributions to parenting literature.

### Strengths and limitations of the study

The inclusion of maternal grandmother and mother dyads enabled triangulation of data. However, the inclusion of maternal grandmothers in the sample might have created a bias in the findings, as maternal grandmothers were mostly mentioned. The G2 mothers were cognizant that their mothers were being interviewed, and this may have primed their responses to be about interactions with the maternal figure.

The findings presented here originate from a study centered on general parenting practices of Bangladeshi mothers, and protective parenting aspects did not receive a focused exploration. Besides, geographical constraints might have a culturally confined view of aspects of protective parenting. A dedicated study conducted in different geographical regions in Bangladesh has the potential to enrich and broaden the applicability of the findings.

## Conclusions and recommendations

This study explored the intergenerational continuity of protective parenting practices between two generations of Bangladeshi mothers. It also identified several aspects and sources of learning that contributed to shaping protective parenting practices learned from earlier generations and to introducing new practices. Future studies are needed to identify the factors that account for the intergenerational continuity of protective parenting practices within the interaction of other learning sources and influencers in the context of globalization and freedom of information.

This study reports that maternal grandmothers have a prominent role in acquiring protective parenting practices in Bangladesh, although urbanization and globalization have gradually modified traditional cultures and lifestyles [58]. Efforts focused on optimal child development, should include maternal grandmothers for better outcomes. As a recommendation, future studies should consider including fathers, grandfathers, and paternal grandmothers to extend research on the intergenerational continuity of parenting practices. The role of other contributors as well as the extent and mechanism of these elements in intergenerational continuity, should also be further investigated.

## Supporting information

S1 Fig Process model of intergenerational continuity of protective parenting practices. (TIF)

S1 Table Socio-demographic characteristics of participants. (DOCX)

S1 File Inclusivity in global research. (PDF)
