# Supplementary figures and images for: Intergenerational continuity of protective parenting practices in Dhaka, Bangladesh
Source: PLoS One. 2025 Feb 28;20(2):e0300160. doi: 10.1371/journal.pone.0300160 (PMC11870356; doi:10.1371/journal.pone.0300160)

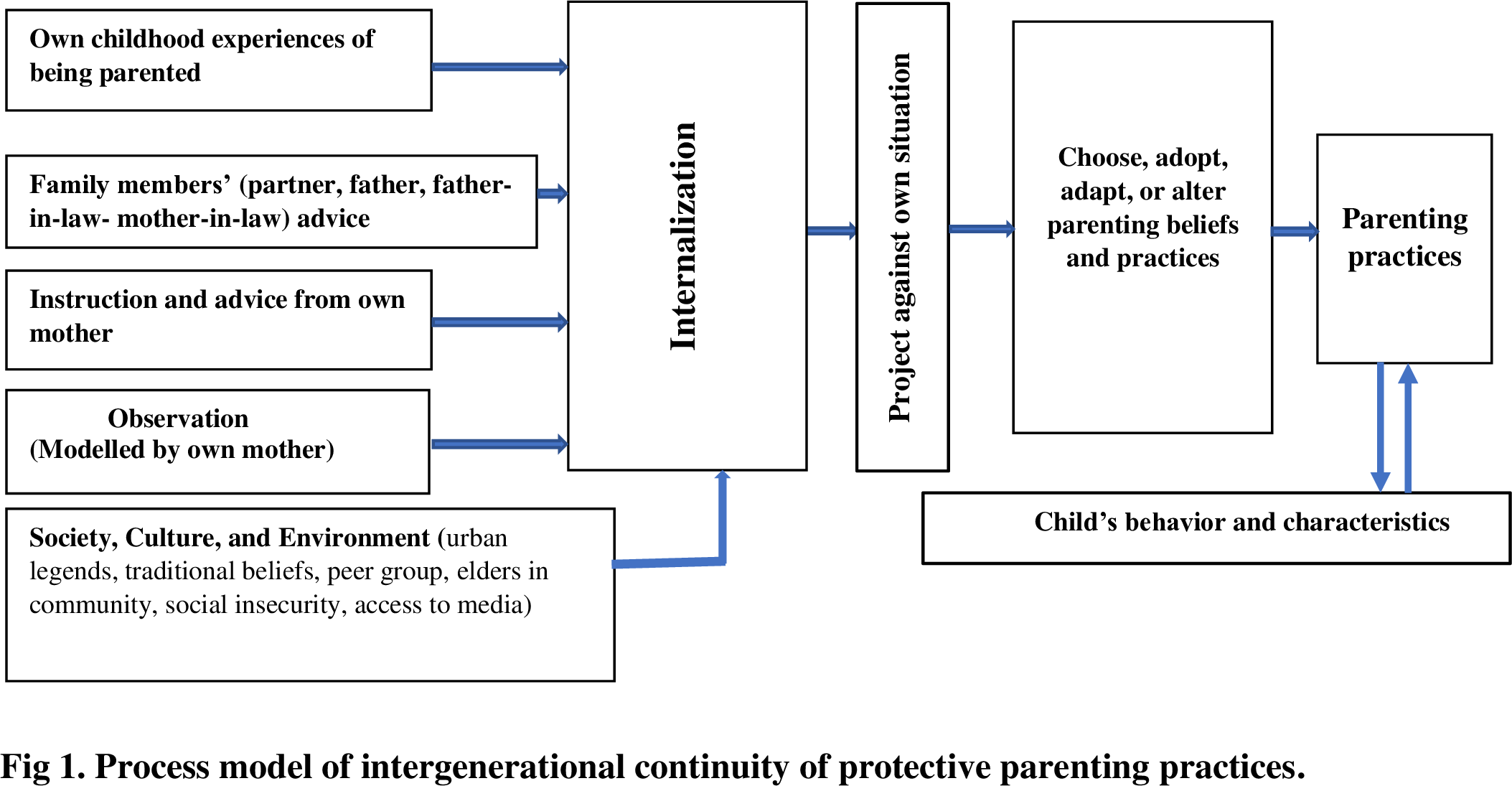

Supplement: S1 Fig — (TIF) [file pone.0300160.s001.tif]
