# Supplementary material for: Intergenerational continuity of protective parenting practices in Dhaka, Bangladesh
Source: PLoS One. 2025 Feb 28;20(2):e0300160. doi: 10.1371/journal.pone.0300160 (PMC11870356; doi:10.1371/journal.pone.0300160)
Supplement: S1 Table — (DOCX) [file pone.0300160.s002.docx]

**Table 1. Socio-demographic characteristics of participants.**

| **Dyads** | **Type of Participants** | **Pseudonym** | **Age**  **in**  **year** | **Highest Education obtained** | **Profession** | **Monthly Family**  **Income (BDT) and sources** | **Number of children** | **Age range of children** |
| --- | --- | --- | --- | --- | --- | --- | --- | --- |
| Dyad # One | Mother | Panna | 42yr | Masters | Housewife | 50,000;  Husband’s business | 1 | 9 yrs |
|  | Grandmother | Tara | 56yrs | Below SSC* | Housewife | 100000; rent and  Husband’s business | 4 | 19-34yrs |
| Dyad # Two | Mother | Dali | 50yrs | HSC** | Housewife | 48,000; rent and Husband’s job | 2 | 18-29yrs |
|  | Grandmother | Hena | 69yrs | Grade 5 | Housewife | 35,000;  Rent | 4 | 33-52yrs |
| Dyad # Three | Mother | Shirin | 49yrs | Masters | Housewife | 40,000;  Husband’s job | 2 | 9-18yrs |
|  | Grandmother | Fatima | 60yrs | Matric | Housewife | 40,000; rent and  Husband’s pension | 3 | 40-52yrs |
| Dyad # Four | Mother | Tonu | 37yrs | Masters | Housewife | 35,000;  Husband’s job | 1 | 12yrs |
|  | Grandmother | Mina | 62yrs | Matric | Housewife | 25,000;  Rent | 3 | 29-41yrs |
| Dyad # Five | Mother | Shoshi | 48yrs | HSC | Housewife | 50,000;  Husband’s business | 2 | 17yrs (twin) |
|  | Grandmother | Rehana | 66yrs | Grade 8 | Housewife | 100,000; rent and  Husband’s pension | 2 | 45-48yrs |
| Dyad # Six | Mother | Pinky | 30yrs | Masters | Housewife | 50,000;  Husband’s job | 2 | 1-7yrs |
|  | Grandmother | Jahan | 49yrs | SSC | Housewife | 40,000;  Rent | 3 | 19-30yrs |
| Dyad # Seven | Mother | Piu | 28yrs | HSC | Housewife | 45,000;  Husband’s job | 2 | 3.5-8.5yrs |
|  | Grandmother | Amena | 48yrs | Grade 5 | Housewife | 32,000;  Husband’s job | 3 | 17-28yrs |
| Dyad # Eight | Mother | Farida | 46yrs | Masters | Advocate | 85,000; rent, profession, and husband’s business | 2 | 14-19yrs |
|  | Grandmother | Jobaida | 80yrs | Grade 5 | Housewife | 35,000;  Rent | 9 | Not available |
| Dyad # Nine | Mother | Charu | 48yrs | Masters | Teacher | 70,000; rent, profession and Husband’s job | 2 | 12-21yrs |
|  | Grandmother | Khadija | 66yrs | < Grade 5 | Housewife | 25,000;  Rent | 2 | 32-48yrs |
| Dyad # Ten | Mother | Dina | 34yrs | Masters | Teacher | 100,000; profession and  Husband’s business | 2 | 6-12yrs |
|  | Grandmother | Sharifa | 51yrs | Below Matric | Housewife | 50,000; Husband’s job/ pension | 4 | 22-34yrs |
| Dyad # Eleven | Mother | Jhumjhum | 36yrs | Masters | Banker | 80,000; job and Husband’s job | 1 | 12yrs |
|  | Grandmother | Kahi | 57yrs | Below Matric | Housewife | 120,000; rent and  Son’s contribution | 5 | 22-38yrs |
